# Sensor-Based Assessment of Soil Salinity during the First Years of Transition from Flood to Sprinkler Irrigation

**DOI:** 10.3390/s18020616

**Published:** 2018-02-17

**Authors:** Mª Auxiliadora Casterad, Juan Herrero, Jesús A. Betrán, Glen Ritchie

**Affiliations:** 1 Unidad de Suelos y Riegos (Associated to CSIC), Av. Montañana 930, 50059 Zaragoza, Spain; acasterad@aragon.es; 2 Estación Experimental de Aula Dei, CSIC, Av. Montañana 1005, 50059 Zaragoza, Spain; 3 Laboratorio Agroambiental, Gobierno de Aragón, Av. Montañana 1005, 50059 Zaragoza, Spain; jbetran@aragon.es; 4 Department of Plant and Soil Science, Texas Tech University, Lubbock, TX 79409-2122, USA; glen.ritchie@ttu.edu

**Keywords:** barley, electromagnetic induction sensor, remote sensing, NDVI

## Abstract

A key issue for agriculture in irrigated arid lands is the control of soil salinity, and this is one of the goals for irrigated districts when changing from flood to sprinkling irrigation. We combined soil sampling, proximal electromagnetic induction, and satellite data to appraise how soil salinity and its distribution along a previously flood-irrigated field evolved after its transformation to sprinkling. We also show that the relationship between NDVI (normalized difference vegetation index) and ECe (electrical conductivity of the soil saturation extracts) mimics the production function between yield and soil salinity. Under sprinkling, the field had a double crop of barley and then sunflower in 2009 and 2011. In both years, about 50% of the soil of the entire studied field—45 ha—had ECe < 8 dS m^−1^, i.e., allowing barley cultivation, while the percent of surface having ECe ≥ 16 dS m^−1^ increased from 8.4% in 2009 to 13.7% in 2011. Our methodology may help monitor the soil salinity oscillations associated with irrigation management. After quantifying and mapping the soil salinity in 2009 and 2011, we show that barley was stunted in places of the field where salinity was higher. Additionally, the areas of salinity persisted after the subsequent alfalfa cropping in 2013. Application of differential doses of water to the saline patches is a viable method to optimize irrigation water distribution and lessen soil salinity in sprinkler-irrigated agriculture.

## 1. Introduction

Salt accumulation occurs in many irrigated systems, particularly in arid regions where the irrigation water is limited and the conflicts for water allocation are intermingled (e.g., [1]). Soil salinity substantially reduces income in irrigated agricultural systems around the world [2,3], as is the case of the Ebro valley, Spain [4,5], where salinity also causes non-point source pollution [6]. Soil salinity is a widespread limiting of the range of crops and their yields. In fields where salt accumulation is a risk, soil sampling for the determination of electrical conductivity or the ionic contents has been traditionally used for identification of salt problems. For agronomic purposes, many authors, as reviewed by Grunwald et al. [7], have stressed the need and limitations of combining data from different proximal or remote sensors with the classical lab- and field-based soil measurements.

Electromagnetic induction (EMI) techniques are popular for mapping soil salinity in agriculture with a substantial reduction of soil sampling sites (e.g., [8,9,10]). EMI techniques rely on instruments—EMI sensors—producing an electromagnetic field with a coil and detecting with another coil a fraction of the secondary electromagnetic field produced by the soil, which depends on its electrical conductivity [8]. Metallic objects, salinity, soil moisture and temperature are some factors affecting EMI measurements. Proximal EMI for agricultural or environmental applications, such as measuring soil salinity on wide areas, may be conducted by mounting the EMI sensor on a mobile device equipped with GPS. This platform enables mapping soil salinity in irrigated lands, allowing wise application of the irrigation water or other inputs. In this context, Triantafilis et al. [11] demonstrated a mobile electromagnetic sensing system to ascertain the cause of soil salinization at an irrigated-cotton farm of 26 ha. Urdanoz et al. [12] reported using mobile and georeferenced electromagnetic sensor measurements to create maps to help select the most suitable crops in a 43 ha saline site and to correlate salinity maps with drainage water quality to determine the salinity-sources in a new irrigated basin.

Another useful tool in detecting and monitoring salt affected soils is remote sensing. Satellite images gather multitemporal information from large areas. However, the detection of the soil salinity in cultivated areas is mediated by the very diverse response of crops to salinity, a diversity that also occurs between the same crop in different areas and even in the same plot along years. Weather and management in the current and precedent years are the main factors of such diversity. The detection of soil salinity by satellite images has been broached with two different approaches: directly, by the spectral response of bare soil, or indirectly, by the type or the condition of the vegetation [13,14,15,16]. In intensively cultivated areas, only the indirect indicators are used for soil salinity detection. Many factors affect crop appearance and yield. The relationship between vegetation spectral indexes and salinity is strong only when salinity is the major growth-limiting factor. Even then, indirect indicators must be carefully checked.

According to the above references, the best results of monitoring the soil salinity by remote sensing are obtained by integrating data from remote sensing with field and laboratory studies. This approach has been applied in irrigated areas with several types of crops. Eldeiry et al. [17] used field measurements with an EM38 electromagnetic sensor plus Landsat and Ikonos images to quantify the variability of soil salinity and minimize the number of samples in their study area. Lobell et al. [18] evaluated the relationships between wheat yield and salinity in an irrigation district using Aster and Landsat images, soil sampling, field salinity measurements with EM38 electromagnetic sensor, and field measurements of yields. Odeh and Onus [19] developed quantitative methods for mapping salinity and sodicity, information essential for the effective monitoring and management of agriculture and soil resources. Other authors used on-site measures of electrical conductivity by a Wenner array as a lump indicator of soil features affecting crop development [20]. In the last years, several authors evaluated the use of very high resolution satellite imagery, such as WoldView-2, for detecting salinity in irrigated areas, and established relationships between the image features and the electrical conductivity of the soil (e.g., Alexakis et al. [21], Muller et al. [22], and Vermeulen et al [23]). Field survey, laboratory analysis, and spectral indices derived from remotely sensed images together with geostatistical and GIS techniques are used to explore and map soil attributes, as is the case at the references in the next paragraph.

Many of these studies were conducted on large areas (e.g., [24,25,26,27,28,29,30,31,32]). Remote sensing images provide current and retrospective spatial-temporal territorial data quickly and regularly. This fact is essential for monitoring the spatial and temporal dynamics of land cover and to know the spatial-temporal trends and changes of areas with salinity. However, the spectral data are affected by a combination of soil salinity, other field characteristics, and input factors, including soil moisture, closely related in dry climate areas with the irrigation amount [33]. Thus, disentangling the relationships between the remotely sensed data and the soil salinity distribution within a plot in irrigated areas requires detailed analysis.

Our work in this article was conducted in a commercial field, representative of the agricultural systems in the irrigated semiarid lands of the central Ebro valley, Spain. The studied field was transformed in 2008 from surface irrigation in small plots to sprinkling irrigation after merging the old plots. One concern when irrigating with sprinklers is the formation of a surface crust [34,35] in the puddles produced when the pluviometry surpasses a determined threshold, as can happen during storms or high irrigation instantaneous volumes. Because of the low EC of the irrigation water, the salts dilution at the surface by rain or irrigation with about 330 mm needed to satisfy the net irrigation requirements of barley (Salvador et al. [36]) can lead to soil dispersion [37], exacerbated by droplets impact, as occurs at the central Ebro valley. Upon drying, the dispersed material produces a crust that hampers both the seed rooting and the emergence of plantlets. These circumstances are common in the irrigated districts of the Ebro valley [38,39,40,41]. Our article is a first step for future appraisals of how soil salinity responds to the change of irrigation technology, and provides a case study about the collection and preparation of soil and ancillary data and their modeling for time-lapse mapping of soil salinity affecting irrigated crops.

This article aims to show how, in 2009 and 2011, the data of proximal EMI sensors, together with a small number of soil samples, enable the measurement and mapping of within-field soil salinity variability and how this map linked with satellite data is a tool to establish the relationship between crop development and soil salinity under sprinkler irrigation. Another purpose is to show that the production function of yield over soil salinity is paralleled by the relationship between NDVI (normalized difference vegetation index) and ECe (electrical conductivity of the soil saturation extracts), a relation rarely established by the literature in irrigated plots.

## 2. Materials and Methods

### 2.1. Study Area

**Figure 1:**
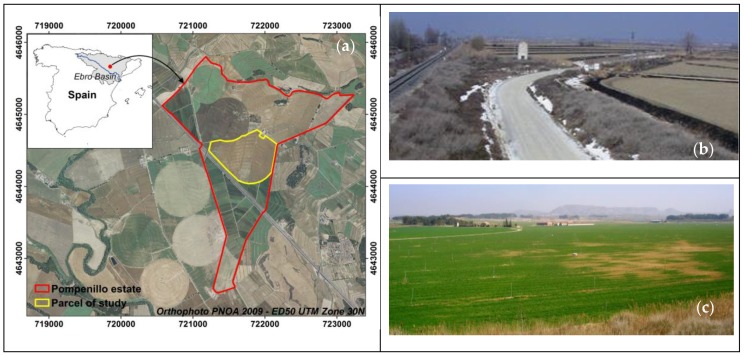
Location of Pompenillo estate (**a**); and examples of salinity problems: on 16 March 2005, white efflorescence in a road and its borders adjacent to just plowed irrigable plots (**b**); and, on 12 March 2009, patches with nil development of barley due to the high soil salinity levels (**c**).

Our study was conducted at the Pompenillo estate, located in the municipality of Grañén, Spain (Figure 1a), within the Flumen irrigation district (between 42°3′18″ N, 0°6′43″ W and 41°43′23″ N, 0°33′34″ W). Based on meteorological data from 2006 to 2014 at the Sodeto weather station (http://eportal.magrama.gob.es/websiar/Inicio.aspx), located 7.2 km away from Pompenillo, the mean annual temperature is 14.2 °C, the mean annual precipitation is 371 mm, with most rains occurring in spring and autumn, and the mean annual potential evapotranspiration is 1210 mm.

The underlying geological material is from the Miocene period, in alternating horizontal strata of sandstone and lutite, often saliferous. The landscape has residual platforms capped by gravelly Quaternary deposits cemented by calcium carbonate. The slopes and bottoms are covered by fine materials composed mainly of silt and illitic clay [42], often organized in alternant millimetric layers forming a varved sediment [43] of silt and sodic clay. In the 1950s, extensive leveling and terracing occurred along with the construction of irrigation and drainage ditches. The soils are Typic Xerofluvents and Oxyaquic Xerofluvents.

**Figure 2:**
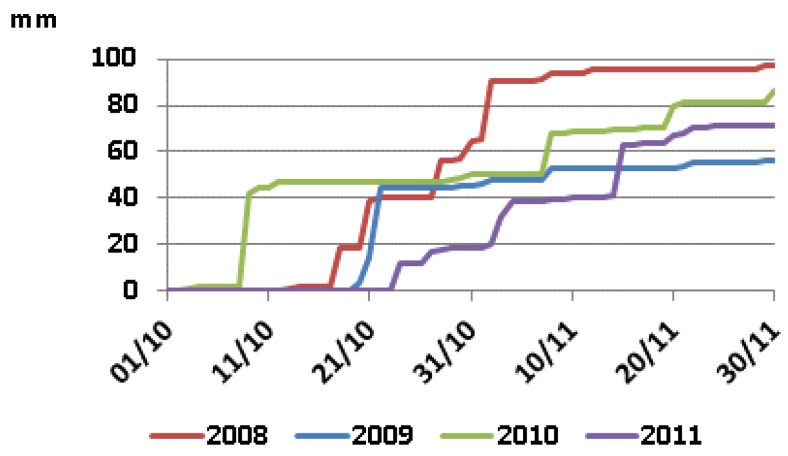
Accumulated precipitation in October and November after the records of the weather station of Sodeto, Spain.

Irrigation started in the 1950s when the Canal del Flumen was built. The irrigation water from the Pyrenees has very low ionic contents, with average EC = 0.23 dS m^−1^, and SAR = 0.3, as reported by Playán et al. [44], at nearby plots in this irrigation district. The leveling and terracing of Pompenillo estate, completed before 1957, resulted in 374 plots typically of <1 ha each, as reported by the late Oficina de Suelos de Huesca [45]. Water was delivered to every plot by individual gates in irrigation ditches built in concrete. The alternating horizontal lutite and sandstone strata make the deep drainage slow favoring lateral transmission and seepage at the escarpments. Soil salinity was not evident when irrigation began, as shown by the cultivation of non-salt tolerant fruit trees: apple, pear and apricot. Agricultural problems due to salinity started a few years after the commencement of irrigation, because the leveling and terracing brought saliferous Miocene materials (e.g., lutite with ECe = 15.4 dS m^−1^ reported by Betrán [45]) to the surface, and later irrigation water redistributed the salts by evapoconcentration at the surface and by lateral transmission. Eventually, the landowner had to uproot the trees and introduce salt-tolerant crops. In the 1980s, most plots of the lowest areas of the farm were converted into rice paddies with continuous flood-irrigation throughout the growing season to avoid the rise of a shallow saline water table. The upper plots of the farm, less saline, were cultivated mainly with barley (*Hordeum vulgare* L.) or other salt-tolerant crops. The average barley yield in Pompenillo estate is 5100–5500 kg ha^−1^ grain. For the present study, we surveyed the soil salinity in 2009 and 2011. Both years had the same management, with barley sown in November and sunflower (*Helianthus annuus* L.) immediately after harvesting barley at the end of June. Rainfall in October and November, just before and after barley sowing in November, is very different from one year to another (Figure 2) and affects the establishment of barley.

Betrán [45] reported ECe values < 2.25 dS m^−1^ with no sodicity at the upper part of the studied field, while the lowest area had ECe > 10 dS m^−1^ and sodium adsorption ratios (SAR) of 15.2 at the surface soil, and a water table at 58 cm depth with EC = 16.34 dS m^−1^ and SAR = 32.9. These data agree with the occurrence of salt efflorescences shortly after windy days following a previous rain episode (Figure 1b), and with the patches with no development of crop whose efflorescences have disappeared due to sprinkling (Figure 1c).

In 2008, the northern portion of the farm was converted to sprinkler irrigation, with new earthwork, to install five center pivots and solid set sprinklers in the corners. Our study area encompassed one of these pivots (35.7 ha) and the adjacent corners (9.5 ha) totaling 45.2 ha (Figure 1a), with elevation ranging from 317.6 m to 313.5 m above sea level and a slope of 0.61% with southern exposure. In 2009, the old plots could still be distinguished in the new sprinkler irrigated plots (Figure 1a). The change from puddle rice to sprinkler irrigation drastically decreased the average irrigation amounts, from 1140 mm to 260 mm for barley and 860 mm for alfalfa [36].

### 2.2. EMI Readings and Acquisitions of Soil Samples

Readings by a DUALEM-1S (DUALEM Inc., Milton, ON, Canada) EMI sensor were carried out using a small tractor towing a non-metallic sleigh bearing a DUALEM-1S. The DUALEM-1S registers EMI signals simultaneously in the vertical transmitter-horizontal receiver mode and the vertical transmitter-vertical receiver mode, achieving 70% of the cumulative response up to depths of 0.5 m and up to 1.5 m, respectively [46]. A correction factor was applied to the readings based on the measured soil temperature to reference them to 25 °C [47], and numbers were divided by 100 for simplicity. The corrected readings of the horizontal and vertical receiving dipole were termed EMh and EMv, respectively. According to the cumulative response in both modes [46], and the targeted 1 m-depth soil, we used for calibration the signal EMh from the dipole in horizontal disposition, i.e., parallel to the ground surface. Data from the towed EMI sensor and from an eTrex Vista GPS unit mounted on the vehicle were stored on an Allegro SX portable computer (Juniper Systems, Inc., Logan, UT, USA) running a HGIS application (Starpal, Inc., Fort Collins, CO, USA) to record the position of the EMI readings [12].

**Figure 3:**
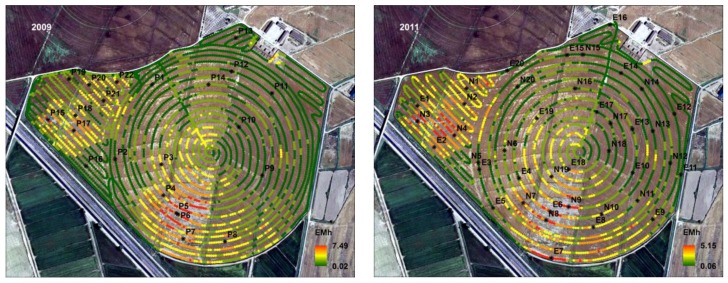
Location of the EMh readings and the soil sampling sites (black dots).

The EMI readings with the mobile equipment were taken in circular tracks at the pivot, and between the rows of sprinklers at the corners (Figure 3). We obtained two maps from the EMI readings while the soil moisture was at field capacity because the soil was trafficable and with sufficient moisture to dissolve the salts. Table 1 shows the number of soil sampling sites, sampling depths, and other details for the two soil survey years. The locations (Figure 3) of the sites for soil sampling by auger were chosen to obtain a balanced distribution of sample sites throughout the EMI readings range and for the area studied. In 2009, a total of 22 sites, P1–P22, were chosen, while, in 2011, we chose 40 sites in two runs, N1–N20 and E1–E20. The sampling sites were re-located the next day with a GPS and new readings with a hand-held DUALEM-1S, followed by soil samples with an Edelman auger. These readings were used for calibration as well as for checking the locations and that the soil conditions did not change. No irrigation or rain occurred between the EMI surveys and the next-day samplings. A water table was not found in any of the auger holes. Soil salinity (ECe and/or EC1:5) was measured in the lab on soil samples selected at random. Gravimetric moisture, texture, and presence of gypsum were measured in 2009.

### 2.3. EMI Readings Calibrations

We were most interested in the salinity of the upper meter of the soil [48], and thus we calibrated our EMI readings to the electrical conductivity of the soil (EC1:5 or ECe) to a 100 cm depth. We used ordinary least squares (OLS) regressions because this popular method has produced good results in previously studied plots before changing to sprinklers [49,50] as well as in other nearby irrigated soils [9,44,51,52,53]. Moreover, the OLS simple computation and interpretation will make easy future routine applications.

As the EMI readings relate to the actual soil moisture, we determined this parameter in 2009 to check its influence on the relationships between the EMI readings and the electrical conductivity of the soil extracts. In the 110 samples taken up to 125 cm depth (Table 1), the soil moisture was determined by putting sub-samples of the soil in sealed aluminum cans that were transported to the lab, weighed during the day, opened and dried at 105 °C and weighted again. The difference of weights was expressed as the moisture content. The presence of gypsum in the soil can also influence the relationship between EMI readings and the electrical conductivity of the soil extracts. The acetone test for detecting calcium sulfate [54,55] by the occurrence or not of a whitish precipitate in the extracts was applied to the 110 soil samples of 2009, and the results classified into three qualitative classes: no gypsum, slight presence of gypsum, and evident gypsum. The reservations of Artieda et al. [56] did not apply, provided that no flocculation or turbidity occurred in the extracts.

### 2.4. Soil Salinity Mapping

Ordinary kriging of the EMh readings was applied to produce the vectorial EMh maps of 2009 and 2011. This method of interpolation, as one of the more flexible and effective, is very used in the literature to obtain salinity maps from EMI measurements. We also tested the inverse distance method, that produced “bull’s-eye” patterns around the EMI reading points. We applied a spherical isotropic semivariogram model using the nearest five points in the interpolation (Range 92.2, Sill 0.41, Nugget 0.043 for 2019, and Range 88.0 Sill 0.35 and Nugget 0.030 for 2011).

We converted maps from vectorial to raster with pixels of 25 m × 25 m to match information from satellites of medium spatial resolution, like Landsat, Spot, or Deimos. ECe was estimated for all EMI reading sites to produce salinity maps referred to the ECe up to 1 m depth. Table 2 shows the equations used for EMh reading calibrations. The ECe map for 2009 was obtained by applying first the regression equation of EC1:5 on EMh (Equation (1)). For this purpose, we calculated an EC1:5 value for each of the 22 sampled sites as the mean of lab-measured EC1:5 in the four samples until 1 m depth. Then, we converted the estimated EC1:5 to ECe by means of Equation (2), obtained by regressing ECe on EC1:5 using the lab determinations of ECe and EC1:5 on 24 soil samples selected at random.

The calculations for 2009 and 2011 were independent and separate. The calculations for 2011 did not involve conversion of EC1:5 to ECe because ECe was determined on all 80 soil samples taken in 2011. ECe was computed for each of the 40 sites sampled as the mean of the ECe of the two soil samples from each site. Then, ECe was regressed directly on EMh, resulting in Equation (3).

The need for calibration for each new field campaign of EMI measurement is an inherent limitation of EMI technology due to the temporal changes in the many factors affecting the intensity of the electromagnetic signal. These factors would need to be considered if a mechanistic approach were used. In most applications of EMI, this need is avoided by using—as we do—a stochastic approach, i.e., calibrating the sensor against the targeted feature, ECe in our case. The equations allowing conversion of the signal into ECe or other expressions of the soil salinity are often different from one date to another, and can be evaluated with standard statistical criteria.

Four salinity phases (Table 3) were drawn in these maps based on the proposal of Soil Survey Division Staff ([57], p. 108).

### 2.5. Vegetative Activity

Vegetation indexes, i.e., combinations or transformations of spectral bands that accentuate the spectral properties of vegetation, are well suited for evaluations of vegetation cover, vigor, and growth dynamics, and are popular in remote sensing agronomic applications. Among the spectral indexes used for salinity detection, the NDVI is one of the most frequently applied. In the central Ebro valley, Amezketa et al. [58] found good correlations between ECe and several spectral indexes, concluding that in the absence of other stressors, NDVI from middle April to early May is an appropriate index for soil salinity characterization in plots planted with barley.

In our study, we used NDVI to determine how soil salinity affected crop development. This index was obtained from Landsat 5TM images of the 199/031 scene acquired in 19 April 2009 and 25 April 2011, free of clouds, when barley attains maximum vegetative development and the NDVI differences between zones with good crop development and zones with poor or nil development are highest. Before the calculation of NDVI, the images were corrected for geometry and radiometry, and resampled to a 25 m × 25 m pixel size. The salinity and NDVI were combined using GIS tools to obtain for each pixel the information shown at the Results section. Pixels located on the borders of the studied area were disregarded because their spectral signatures are often a mixture of two or more ground covers, and then their NDVI would be not representative of the target crop. A zonification of the vegetative activity for the studied plot was obtained by unsupervised classification using the NDVI images from 2009 and 2011. The NDVI signature for each class obtained was analyzed and interpreted according NDVI values to group the classes discriminated with similar behavior and to stabilize the four final categories: Poor or nil, Middle, Good, and Very good vegetative activity. Classifying vegetative activity in four groups parallels the four salinity phases, with names for the groups coherent with the saline phases: Very good–Non saline, Good–Moderately saline, Middle–Strongly saline, and Poor or nil–Very strongly saline.

## 3. Results

### 3.1. Soil Salinity

In 2009, a positive and significant (*p* < 0.01) relationship of EMI readings with soil salinity was found, but not with soil moisture. This was as expected in the two years, due to the homogeneous soil moisture at the time of EMI measurements. Both the nature of the parent material and the earth movements in the 1950s and later in 2008 point to a rather homogeneous texture of the studied soil, which is confirmed by the homogeneity of the water content. Gypsum was not found in the visual inspection of the 110 soil samples, and its content was negligible after the acetone test, with slight precipitate in sox samples, and evident precipitate in two samples.

**Figure 4:**
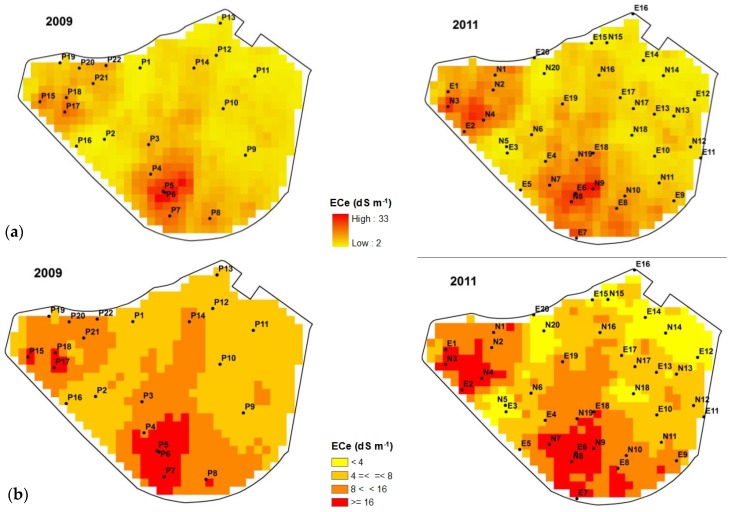
Salinity maps (**a**); and their classification in four saline phases (**b**) for each year studied.

The saline patches are almost coincident in both years, with the most saline areas at the south and west sides of the plot (Figure 4). Salinity increases downslope, with the lowest areas (317.6 m a.s.l.) suffering severe and recurrent salinity stress. Only small differences occurred when comparing the extent in 2009 and in 2011 of the area with salinity problems for barley cropping, i.e., over a threshold of 8 dS m^−1^. In 2009, 55.1% of the surface is below the mentioned threshold and 52.4% in 2011 (Table 2). The encroachment of the very strongly saline soil from 8.4% to 13.7% of the total surface area suggests that the salinity increased in the already affected areas.

### 3.2. Vegetative Activity

**Figure 5:**
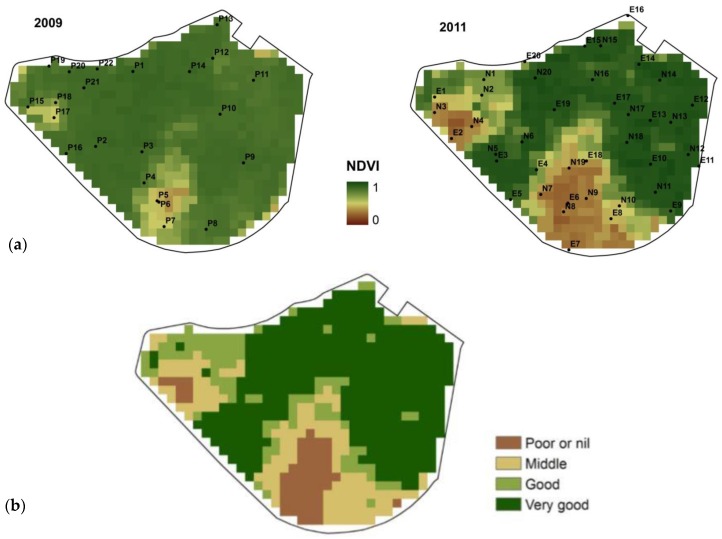
Vegetative activity in April for each year studied (**a**); and zonification of vegetative activity (**b**).

The spatial pattern of vegetative activity for 2009 and 2011 agrees with the spatial distribution of salinity in the plot, with the most saline areas (Figure 4) showing the lowest values of NDVI, indicative of the worst vegetative development in those areas (Figure 5). The distribution of the areas showing bad or nil plant development either in the NDVI images or in the field visits (Figure 1c) were not related with the sprinkler irrigation system or with visible soil properties others than salt-affection. The highest differences in the relationship between NDVI and ECe (CEe/NDVI) from 2009 to 2011 occurred in the strongly and very strongly saline patches where the development of barley is heavily conditioned by the soil salinity. Not relevant differences occur in the rest of the pivot, where ECe is under the threshold of barley tolerance to salinity (ECe = 8 dS m^−1^).

From the NDVI obtained for soil sampling sites, we discriminated four groups of behavior according to the vegetative development and activity (Table 4). The vegetative activity in 2011 was lower than in 2009 for all groups, except Group A. Groups A and B had similar values and inter-annual trends for NDVI, with narrow ranging. The small variability in these groups contrasts with Groups C and D, where crop development is problematic. NDVI at the sites belonging to Groups C and D shows a broad range, and occasionally the trends of NDVI were quite dissimilar within the same groups. The highest variability occurs at Group B. These four groups of behaviors allow classifying the field by the vegetative activity (Figure 5), which is related to soil salinity, as discussed below.

**Figure 6:**
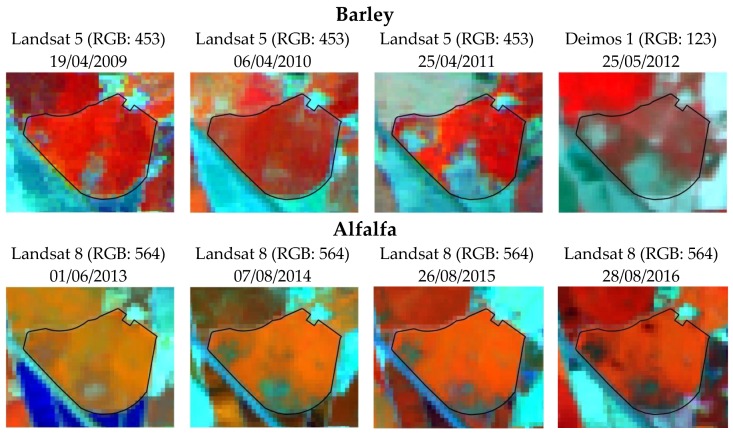
False color compositions from satellite images of the studied field.

Vegetative activity continues to be affected by the salinity eight years after the beginning of sprinkler irrigation, despite the change to alfalfa, a moderately salinity-tolerant crop [59,60,61] sown at the end of winter season. The satellite images in Figure 6 show the persistence of salinity during this period. The blue colors in false color compositions denote areas with poor or nil vegetative activity while red colors denote active vegetation, with higher red intensity indicating more vegetative activity. Alfalfa is a perennial crop with permanent ground coverage throughout its life cycle, reducing the capillary ascent of salt. Furthermore, the alfalfa in the region is well irrigated. However, the areas with poor or no crop development had the same location in all years, with the extent of salt stress varying among years in the high salinity patches. The alfalfa images shown in Figure 6 were chosen when the coverage of soil is highest, i.e., at the end of crop development or at mid-season phenology stage. The NDVI values obtained from these images confirm the persistence of salinity problems. NDVI values are high, 0.86, at the zones without salinity due to the very good development and coverage of the crop, while the NDVI values decreased to as low as 0.48 for zones with severe salinity.

### 3.3. Relationship between Vegetative Activity and Soil Salinity

**Figure 7:**
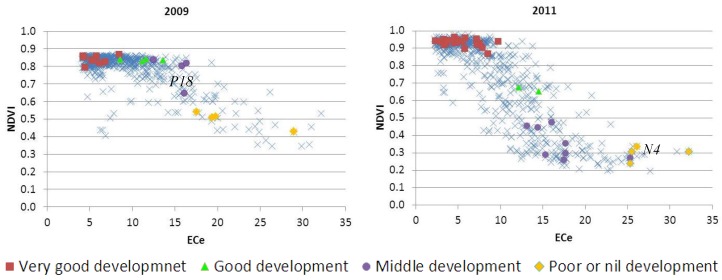
Scatter diagram of NDVI in April versus ECe (dS m^−1^) for the years 2009 and 2011. Each cross represents a pixel, with the sampling sites highlighted in colors according to their vegetative activity.

The relationship between NDVI and ECe is similar for the two years studied. In both years, barley has good vegetative activity and development until a soil salinity threshold, and then the vegetative activity decreases as soil salinity increases until a nil activity for high salinities (Figure 7). The scatter diagrams of NDVI versus ECe (dS m^−1^) at each pixel for the years 2009 and 2011 indicate a two-stage process at pre- and post-threshold salt concentration: no effect at pre-threshold concentration and a linear decline with increasing salinity at post-threshold. However, the scatter diagrams are somewhat different. The range of NDVI in 2009 is smaller than in 2011. In 2009, the pre-threshold concentration part of diagram is wider than 2011 and the slope of post-threshold points lower. In addition, in 2011, the nil vegetative activity (NDVI around 0.2) was found in several pixels.

For the interpretation of the scatter diagrams, the following circumstances must be taken into account: (i) the size of the pixel (25 m × 25 m); (ii) the location procedure for the sampling sites with a metric GPS having an error between 5 m and 10 m; and (iii) the fact that some of the sampled sites fell in the border zone between two saline phases. An example is site N4 in 2011 (Figure 4) belonging to Group B, whose map-estimated ECe is 25.3 dS m^−1^ against 15.6 dS m^−1^ determined at the lab, an ECe that would locate this site together with the other sites of Group B. In the same way the site N4 appears in 2011 with the poor or nil development group in Figure 7.

Another example is the site P18 in 2009, located in the middle of the slope in Figure 7. For this location, the lab-measured ECe was 18.4 dS m^−1^, compared with a similar map-estimated ECe of 16.1 dS m^−1^. The location of P18 at the border between two saline phases produces the peculiar behavior. The site P18 had in 2009 lower NDVI value than the other Middle development sites (Figure 7). Moreover, for P18 the difference between NDVI in 2009 and NDVI in 2011 was 0.18, less that the others 11 sites of Group C-Middle development with differences ranging from 0.33 to 0.53. In the border areas between the phases Strongly saline and Moderately saline, the development of barley, a salt-tolerant crop [48], is dependent upon a subtle variation in salinity from one year to another.

The four groups established according to vegetative activity (Figure 5) are related to soil salinity (Figure 4). In Figure 7, the sampled sites are highlighted with different colors depending on the group by behavior (Groups A, B, C, or D) they belong to. The sites classified as Group A, Very good development, fall in the upper plateau, while the sites belonging to Group D, Poor or nil development, fall in or close to the lower plateau. The location of the other two groups, Groups B and C, changes from one year to the other, but they commonly are located on the slant zone, between the upper and the lower plateaus; Group B, Good development, is closer to the upper plateau, while Group C, Middle development, is closer to the lower plateau. In 2009, the pixels from Groups B and C are close to the elbow linking the upper plateau with the slope, whereas, in 2011, they are distributed throughout the slant zone.

**Figure 8:**
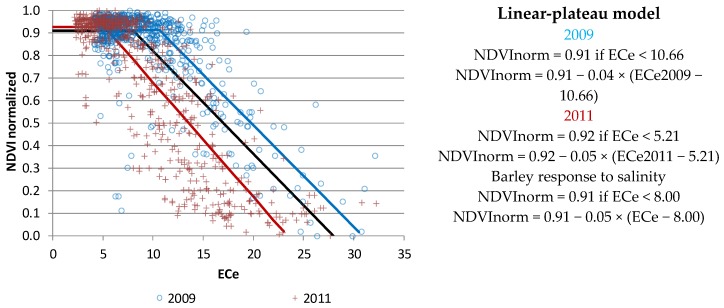
Salinity–NDVI linear response plateau model for barley in 2009 (blue line), 2011 (red line), and function of barley production response to salinity adapted from Ayers and Westcot [48] (black line).

The scatter diagrams of NDVI and ECe at each pixel for the years 2009 and 2011 (Figure 7) mimic the production function between yield and soil salinity proposed by Ayers and Westcot, [48]. In our study, this agreement is illustrated in Figure 8, where NDVI values are normalized (Min-Max standard method) and a linear response plateau model (LRP model) has been used to describe NDVI response to soil salinity. In addition, the barley production responses to salinity are figured (Figure 8 black line) with the NDVI plateau value as NDVI normalized average for ECe < 8 dS m^−1^ and nil production at dS m^−1^ according to Ayers and Westcot [48]. The three models give a maximum NDVI normalized value around 0.91 and the critical value from which the vegetative activity is affected by salinity ranges from ECe 5.2 dS m^−1^ in 2011 to 10.6 dS m^−1^ in 2009.

## 4. Discussion

### 4.1. Vegetative Activity and Its Relationship with Soil Salinity

The herein presented methodology has proven to be suited for mapping the saline phases and delineating zones of vegetative activity. Our results demonstrate that the information of soil and crops gathered with the proximal EMI sensor and the remote TM and OLI sensors on board of Landsat satellites can be used for mapping soil salinity and vegetative activity in a cultivated plot. These maps allow establishing and analyzing the relationships, numerical and spatial, between these two variables, with an accurate spatial matching of both information layers as a requirement for reliable results. The greater variability in the relationship between salinity and vegetative development occurs in the transitional fringes between areas of different degrees of salinity and/or vegetative development, making the cartographic matching especially relevant in these areas. Using images with greater spatial resolution would allow a more detailed delineation of patterns but with an increase of costs of imagery—Landsat and Sentinel images are free—together with an increase in the volume of processed data. On the other hand, very detailed images not forcefully imply better results, and may imply only more noise in the information to be processed. The precision of the GPS in locating the sampling sites is also a concern for the accurate assignation of the pixel.

In this study we compared the vegetative activity and its relationship with soil salinity against the relationships between crop production and soil salinity proposed by Ayers and Westcot [48] described in the literature either as a piecewise linear function [59] or as a sigmoidal function [60], as reviewed by [61]. Grieve et al. [62] stated that “salt tolerance of a crop can be described as a complex function of yield decline across a range of salt concentrations” and stressed the difficulties for establishing such thresholds from experimental data, and their uncertainty. The piecewise function, probably the most popular, is based on a threshold ECe that causes the initial reduction in the maximum expected yield, and the straight line with a slope equaling the ratio between yield reduction and the increase of salinity above the threshold value. This line links an upper line for the maximum production until an ECe value causing nil production, with another horizontal straight line. The threshold value of ECe for barley is 8 dS m^−1^, with yield decreases of 10%, 25% and 50% for ECe of 10 dS m^−1^, 13 dS m^−1^, and 18 dS m^−1^, respectively, and 28 dS m^−1^ for zero or no barley development [48].

The relationship shown in Figure 7 does not allow estimating parameters like those presented for production versus salinity by Ayers and Westcot [48]. For this purpose, images from the same development stage would be needed, but the development of the crop is linked to weather, variable from year to year. Moreover, agronomical variables, including soil salinity, crop cultivar, sowing date, cultural labors, or irrigation management, influence the vegetative activity and crop development. The normalization of NDVI values is necessary to study the agreement of NDVI and ECe relationship obtained in 2009 and 2011, and its similarity with the typical function of barley production response to salinity. The 2009 and 2011 LRP models obtained in our study area (Figure 8) are similar to those relating crop production [63] or germination [64] of barley with salinity in experiments performed at the Ebro valley.

For Ayers and Westcot [48] zero yield potential is at 28 dS m^−1^, the theoretical soil salinity (ECe) at which barley growth ceases. In our case, NDVI normalized equal zero could not mean zero vegetative activity, because in our normalization the minimum NDVI value in the study area has been translated to zero, i.e., the ECe value for NDVI normalized equal to zero is the minimum vegetative activity in the study area and year, which could be associate to minimum production. In the same way, plateau NDVI normalized value corresponds to maximum vegetative activity and maximum production.

### 4.2. Integration in Agricultural Management

The information obtained together with the meteorological data allows better understanding the relationship between soil salinity and agricultural management in the study area. The broadest extent of the crop coverage happened in 2009 and 2010, contrasting with 2011 and 2012, when the surface of non-developed crop was much higher (Figure 6). Rainfall in October and November of 2008 and 2009 was very different from the same months of 2010 and 2011 (Figure 2), just before and after the barley sowing in November. Rains before sowing can leach soil salts, while rains after sowing can produce surface crusting in the saline-sodic patches, leading to poor seedling emergence. This is a well-known problem in the area, designated as “encarado” by the local farmers.

A heavy rain would produce soil supersaturation or inundation in patches because of the low infiltration capacity due to fine textures and sodicity reported for this field by Betrán [45] at the lower area where the spots with undeveloped barley occur, with SAR = 15.2, i.e., surpassing the threshold of SAR = 13.0 for sodic soils. Moreover, the impact of rain or sprinkler drops exacerbates the dispersion of soil aggregates [65] at the surface of the bare patches, and upon drying the dispersed material forms a crust creating impedance for the emergence of plants. These low areas having in 1985 a saline-sodic water table at 58 cm depth [45] remained for years planted with rice in paddies submitted to continuous flooding with running irrigation water, a common setting at the saline-sodic soils of Flumen irrigation district [44,52].

In October 2008 and 2009, the precipitation patterns were similar, with most of the rainfall occurring before sowing and insignificant rainfall afterwards (Figure 2). The amount of rain was 52.9 mm during 18–28 October 2008, with 25.3 mm more on 2 November, while 44.7 mm fell between 20 and 22 October 2009. The rains just before sowing favored the leaching of salts, and the insignificant amount of rain in November of both years did not produce crusting. These conditions favoring the barley emergence explain the high crop coverage in 2009 and 2010. In October 2009, the precipitation was somewhat lower than in the same month of 2008, but the barley of 2009–2010 was preceded by sunflower in the summer of 2009. The more continued soil coverage by plants achieved in 2009 contributed to lessen the ascent of salts during the summer. This would result in more favorable conditions for germination at the patches of high salinity in November 2009 when the barley was sown, provided that the highest sensitivity of barley to salinity occurs at the germination stage. In 2010, the NDVI was >0.43 at all the pixels of the field studied, with more plant covered surface than in 2009.

Contrasted with October–November 2008 and 2009, the same months of 2010 and 2011 had much lower precipitation in the last two weeks of October and higher precipitation in November (Figure 2). October 2010 accumulated 49.0 mm rain, an amount similar to the same month of 2009, but 40.0 mm fell on 9 October well before sowing. In 2011, 18.2 mm fell from 24 to 28 October, with 12.4 mm in 24 October. Two relevant rain events occurred in November 2010: 17.4 mm on 8 November and 9.8 mm on 20 versus a total rain of 61.4 mm in 2011, with 19.8 mm between 2 and 4 of November with a relevant event of 22.2 mm on 15 November. These weather features were unfavorable for plant emergence because of the insignificant salt leaching and the soil saturation and crusting due to the rains in November.

The worst coverage and development of barley occurred in 2012 (Figure 6) because of poor emergence, plus winter drought, as shown by the 110.8 mm of precipitation which fell from 1 October 2011 to 31 March 2012 versus 175.6 mm, 190.3 mm and 234.4 mm during the same period of 2008–2009, 2009–2010 and 2010–2011, respectively. Sunflower, which was planted in the summer of 2011, did not improve the barley emergence.

The satellite images from 2009 to 2012 display the spatial-temporal variability of the vegetative activity of the barley, heavily affected by salinity at the germination and emergence stages. However, the yield data of 2010 show production of <1000 kg ha^−1^ of grain at sites with the highest salinities after the maps obtained with EMI in 2009 and 2011. The image of 2010 (Figure 6) does not show severe salinity problems despite this low yields. It means that crop coverage for the whole was acceptable, but the production was very low or nil at the saline areas, probably due to lodging, to failure of the grain filling, or to the development of volunteer halophytes not discriminated from barley at the images, or to a combination of these circumstances.

The rain at the preceding and immediately following barley sown is the main determinant for crop emergence. Planting two crops, barley followed by sunflower, seems sound for controlling topsoil salinity in summer. As both soil salinity and the vegetative activity can be georeferenced, this information could be used together with digitized fine topography in precision irrigation to combat the effects of salinity. The availability of variable-rate irrigation control systems [66,67] increase the feasibility of differential irrigation at the most saline spots, which could result in saving irrigation water and improving productivity. Transitioning from paddy rice cultivation to barley and alfalfa may require gypsum amendment to combat soil sodicity. However, many farmers are reluctant to do so because of previous failures of this method in nearby areas with similar soil parent material consisting of varved Quaternary sediment [43].

Provided that satellite images respond to the vegetation status, which can result from several factors related to soil salinity, the EMI maps are concordant and seem sufficient [20] to be combined with the remote sensing for crop development watching. However, the knowledge of the causal factors of deficient crop development, salinity in our case, is needed to design and apply remediation measures. For this purpose, the available legacy soils data combined if necessary with collected field data [68] will be a key tool. Georeferencing soil salinity and vegetative activity enables site specific monitoring, management, and salinity control.

In summary, the soil salinity maps of the studied field in 2009 and 2011 show the location of saline spots and their degree of salinity at the first years of the transformation from basin and border irrigation to sprinkling. The interpretation of NDVI and false color images from remote sensing for 2009 to 2016 denote the permanence of the spots with deficient crop development, even after the crop change from the rotation of barley/sunflower to alfalfa. The herein presented information, soil salinity maps and remote sensing data, will be a baseline for future appraisals of the performance of sprinkler-irrigated crops for soil salinity abatement. Moreover, the observed agreement of the relationship NDVI vs. ECe, and yield vs. soil salinity could be exploited to assess the yield variability due to salinity. This information would be very useful for implementing precision agriculture.

Salinity maps obtained with lapses of several years can be overlapped with maps of vegetative activity made for each year from free-available data of Landsat or other medium-resolution satellites. This combination will help to improve soil management, especially at sprinkler-watered fields, where precision irrigation is feasible. It would be a bone for saving irrigation water and diminishing the saline outflows to the rivers and sub-surface waters. The availability of georeferenced mobile systems for EMI studies have expedited the soil salinity mapping with a reduced number of soil samples. The combination with satellite data seems now feasible for big farms or even for whole irrigation districts opening the possibility of tailored applications, including the long term appraisal of sprinkler and precision irrigation for the desired desalination of soils.

Our work demonstrates the evolution of salinity regions in a field during the first years (2009–2011) of the transition from flood to sprinkler irrigation, as well as the persistence of salinity despite the change. This information will help to improve the use and management of irrigated plots threatened by salinity, and to track the effects of the aspersion irrigation on soil salinity.

## Figures and Tables

**Table 1:** Total number of sites of EMI readings, sites by ha, and % inverted profiles (EMv < EMh); number of soil sampling sites, depth, sampling depth interval, and number of samples.

Date of the Survey	Sites of EMI Reading			Soil Sampling			
	Total	by ha	EMv < EMh, %	Number of Sites	Depth cm	Sampling Depth Interval, cm	Number of Samples
12 November 2009	3651	81	3.7	22	125	25	110
21 June 2011	2716	59	20.1	40	100	50	80

**Table 2:** Regression equations with their coefficient of determination (*R*^2^), standard error (*S*), and the number and kind of observations used in the regressions Total number of sites of EMI readings, sites by ha, and % inverted profiles (EMv < EMh); number of soil sampling sites, depth, sampling depth interval, and number of samples.

Year			*R*^2^ %	*S* dS m^−1^	Observations	
					Number	Kind
2009	Equation (1)	EC1:5 = 0.46 + 0.50 × EMh	87.7	0.35	22	Sites
	Equation (2)	ECe = −0.41 + 9.20 × EC1:5	90.2	3.64	24	Soil samples
2011	Equation (3)	ECe = 1.35 + 7.75 × EMh	94.1	2.42	40	Sites

**Table 3:** Salinity phases for soils, derived from Soil Survey Division Staff [57], and their percent extent in the two years studied.

ECe, dS m^−1^	Salinity Phases	Percent Surface	
		2009	2011
ECe < 4	Non-saline or Slightly saline	0.0	17.4
4 ≤ ECe < 8	Moderately saline	55.1	35.0
8 ≤ ECe < 16	Strongly saline	36.5	33.9
16 ≤ ECe	Very strongly saline	8.4	13.7

**Table 4:** Statistics of the four vegetative development groups (Very good, Good, Middle, and Poor or nil) established according to NDVI.

	Group A		Group B		Group C		Group D	
	Very Good		Good		Middle		Poor or Nil	
	2009	2011	2009	2011	2009	2011	2009	2011
No. of samples	32	32	6	6	12	12	9	9
Max NDVI	0.87	0.96	0.85	0.80	0.85	0.51	0.54	0.34
Min NDVI	0.79	0.85	0.83	0.65	0.64	0.26	0.36	0.20
Range	0.07	0.11	0.01	0.14	0.21	0.25	0.18	0.14
Median	0.84	0.93	0.84	0.68	0.80	0.33	0.46	0.29
Mean	0.84	0.93	0.84	0.70	0.77	0.37	0.47	0.28
Standard deviation	0.02	0.03	0.01	0.05	0.07	0.09	0.06	0.05
Coefficient of variation	2.46	2.93	0.65	7.42	9.70	25.05	13.32	17.93
